# BG-YOLO: A Bidirectional-Guided Method for Underwater Object Detection

**DOI:** 10.3390/s24227411

**Published:** 2024-11-20

**Authors:** Ruicheng Cao, Ruiteng Zhang, Xinyue Yan, Jian Zhang

**Affiliations:** 1 School of Cybersecurity, Northwestern Polytechnical University, Xi’an 710072, China; caoruicheng@mail.nwpu.edu.cn; 2 College of Computer Science and Technology, Zhejiang University, Hangzhou 310027, China; 3210104884@zju.edu.cn; 3 College of Computer Science, Chongqing University, Chongqin 400044, China; 202414021035t@stu.cqu.edu.cn; 4 School of Tropical Agriculture and Forestry, Hainan University, Haikou 571158, China

**Keywords:** underwater object detection, underwater image enhancement, feature guided, joint optimization

## Abstract

Degraded underwater images decrease the accuracy of underwater object detection. Existing research uses image enhancement methods to improve the visual quality of images, which may not be beneficial in underwater image detection and lead to serious degradation in detector performance. To alleviate this problem, we proposed a bidirectional guided method for underwater object detection, referred to as BG-YOLO. In the proposed method, a network is organized by constructing an image enhancement branch and an object detection branch in a parallel manner. The image enhancement branch consists of a cascade of an image enhancement subnet and object detection subnet. The object detection branch only consists of a detection subnet. A feature-guided module connects the shallow convolution layers of the two branches. When training the image enhancement branch, the object detection subnet in the enhancement branch guides the image enhancement subnet to be optimized towards the direction that is most conducive to the detection task. The shallow feature map of the trained image enhancement branch is output to the feature-guided module, constraining the optimization of the object detection branch through consistency loss and prompting the object detection branch to learn more detailed information about the objects. This enhances the detection performance. During the detection tasks, only the object detection branch is reserved so that no additional computational cost is introduced. Extensive experiments demonstrate that the proposed method significantly improves the detection performance of the YOLOv5s object detection network (the mAP is increased by up to 2.9%) and maintains the same inference speed as YOLOv5s (132 fps).

## 1. Introduction

**Figure 1:**
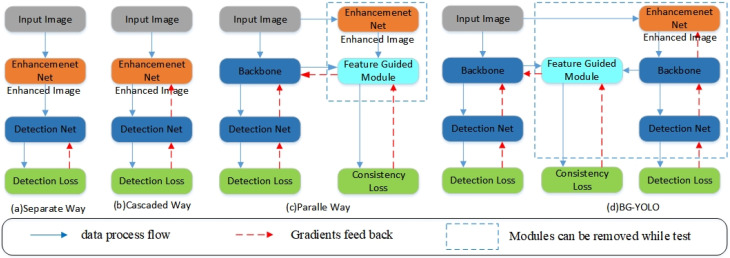
Different ways of combining underwater image enhancement and object detection.

In underwater scenes, images suffer from wavelength-related light absorption and scattering, which results in serious degradation and impairs the accuracy of underwater object detection tasks. Some studies have attempted to address this problem and improve detection accuracy by using underwater image enhancement methods to improve the quality of degraded images. However, image enhancement tasks and object detection tasks have different goals and indicators, which leads to differences in the optimization studies and optimal solutions [1]. Therefore, adopting image enhancement as a direct pre-processing method may not effectively improve the accuracy of an object detection model [2]. Many researchers have begun to focus on combining underwater image enhancement networks and object detection networks to improve the accuracy of underwater object detection. Liu et al. [3] classified the combinations of these as follows—the separate way, the cascaded way, and the parallel way—as shown in Figure 1a–c.

As is shown in Figure 1a, the separate method introduces image enhancement as a pre-processing step before object detection. In this method, image enhancement and object detection are regarded as two individual tasks to be optimized separately, and the enhanced images are used as a training dataset to train the object detection network. This widely used combination is simple and easy to implement. However, considering that image enhancement and object detection are two individual tasks with different optimization indicators, adopting image enhancement as a pre-processing step often does not achieve intuitive improvements in detection tasks [2,4].

In the cascaded method (Figure 1b), the image enhancement network and object detection network are integrated into a single pipeline, which ensures that the two individual tasks are optimized in a common direction through joint optimization. In DE-YOLO [5] and the literature [6], an image enhancement module and detection module are integrated into a single framework in a cascaded manner, and relevant detection information from the detector is used to guide the optimization of the enhancement module in a direction that benefits the detection task, improving the accuracy of object detection. Organizing the two networks in this cascaded manner improves the performance of the detection tasks but introduces an additional computational cost in the test stage.

Different from the two methods mentioned above, in the parallel method (Figure 1c), the image enhancement network and object detection network are organized in a parallel manner; enhanced images are used to guide the training of the detection network to improve the performance of object detection in various degraded scenes [7,8]. Liu et al. [3] organized the enhancement branch and detection branch in a parallel manner and introduced a feature-guided module guiding the shallow layers of the detection branch to learn the lost details of objects using enhanced images. During the test stage, the enhancement branch and feature-guided module are removed, so no additional computational cost is introduced. In contrast to the cascaded method, in the parallel method only the detection branch is reserved, so no additional cost is introduced. However, Liu et al. adopted an individually trained enhancement network, which could not ensure that the images used for guidance were always conducive to the object detection tasks.

In response to the limitations mentioned above, we attempt to combine the advantages of the cascaded and parallel method and propose a bidirectional-guided underwater object detection method, referred to as BG-YOLO (Figure 1d). BG-YOLO consists of an image enhancement branch, object detection branch, and feature-guided module. Specifically, the image enhancement branch and object detection branch are organized in a parallel manner, and the feature-guided module connects their shallow convolution layers, optimizing the network’s training direction by constraining the low-level features of both branches. Our proposed method is significantly different from the parallel method above proposed by Liu et al. [3]. First, in Liu et al.’s method, the image enhancement branch uses a trained network, which is not always conducive to detection tasks. In contrast, in our proposed method, object detection is used to guide the training of the image enhancement branch, optimizing it in a direction conducive to object detection. Therefore, our method has better generalization ability. Additionally, Liu et al. used enhanced images to constrain the training of the detection branch through consistency loss. However, the enhanced image output from the enhancement branch has essential differences from the shallow feature map from the detection branch. In contrast, we constrain the training of the detection module using the consistency loss between the low-level features of the two branches.

To summarize, our main contributions are as follows:

- We propose an object detection framework in underwater degraded scenes, BG-YOLO. Firstly, the detection tasks are used to guide the training of the image enhancement network, which makes the enhancement network conducive to detection tasks. Subsequently, the image enhancement branch and object detection branch are organized in a parallel manner, and the image enhancement branch is used to guide the training of the object detection branch. Finally, during the detection, only the object detection branch is reserved; thus, no additional computational cost is introduced.
- We imposed constraints on the corresponding convolutional layer of the image enhancement branch and object detection branch, both of which have the same dimensions and underlying semantics. This enables the detection branch to learn more feature information, thereby improving its object detection performance.
- Extensive experiments on URPC2019 and URPC2020 demonstrated that our proposed BG-YOLO significantly improves detection performance compared to the original detection method.

The rest of this article is organized as follows: Section 2 briefly reviews underwater image enhancement and the related existing techniques. Section 3 provides a detailed exposition of the proposed method. Section 4 presents the experiments. The conclusion is presented in Section 5.

## 2. Related Work

The objective of this research was to improve the performance of object detection in complex underwater environments, which mainly involves techniques relevant to underwater image enhancement and object detection. We first review the existing research findings in the field of underwater image enhancement. Then, we briefly illustrate the progress of object detection techniques in complex underwater environments. Finally, we focus on the joint optimization of underwater image enhancement and object detection tasks.

### 2.1. Underwater Image Enhancement

Underwater image-processing techniques can overcome the problems of image degradation to a large extent. Underwater image enhancement techniques are classified into traditional approaches and deep learning-based approaches.

Traditional underwater image enhancement approaches include non-physical model-based methods [9,10,11,12,13,14,15,16,17,18] and physical model-based methods [19,20,21]. With the wide application of deep learning in various computer vision tasks, deep learning-based algorithms have been applied to underwater image enhancement, achieving remarkable results [22,23,24,25,26]. Li et al. [27] proposed UWCNN, a convolutional neural network model for underwater image enhancement based on an underwater image prior, which directly restores clear underwater images. Espinosa et al. [28] combined the discrete wavelet transform (DWT) and proposed a variant of U-Net for underwater image enhancement in which the discrete wavelet transform is utilized in skip connection and is used to achieve de-blurring and color correction with the channel attention module. Generative adversarial networks (GAs) are also widely applied to underwater image enhancement [29,30,31]. Jiang et al. [32] proposed a domain adaption framework for real-world underwater image enhancement using CycleGAN [33] to convert underwater-style images into in-air-style images, thereby improving their quality. Image enhancement methods based on deep learning can obtain enhanced images with vivid visual effects without estimating prior parameters but require a large amount of paired data to train the networks.

### 2.2. Underwater Image Detection

With the development of deep learning technology, object detection algorithms based on deep learning have become widely used in underwater object detection [34,35,36]. Zeng et al. [37] introduced an adversarial occlusion network (AON) into Faster R-CNN, effectively preventing overfitting through adversarial learning and consequently achieving a more robust detection network for underwater object detection. Cao et al. [38] utilized lightweight MobileNetv2 as the backbone network of the SSD algorithm and proposed Faster MSSDLite for underwater detection tasks involving live crabs. Liu et al. [39] utilized YOLOv4 as the backbone network, using a dual-branch structure of a detection branch and tracking branch in a parallel manner to detect and track marine fish in real time. Yu et al. [40] designed an underwater object detection network, U-YOLOv7, based on YOLOv7 to meet both speed and precision requirements. In underwater scenes, the distortion of images is the main factor affecting the performance of object detection. Image enhancement can intuitively improve the visual performance of underwater images. Effectively combining image enhancement algorithms to improve the performance of object detection in low-quality underwater images remains a research objective with significant value.

### 2.3. Joint Optimization

Recent studies have integrated the tasks of image enhancement and object detection into one end-to-end framework, optimizing both networks jointly during training [1,41,42]. In IA-YOLO [43], a differentiable image-processing module, DIP, is introduced, which uses a small convolutional neural network, CNN-PP, to predict the parameters of DIP and achieve better detection performance through the end-to-end joint learning of CNN-PP and YOLOv3. In DE-YOLO [5], the image enhancement module DENet and the detection module YOLOv3 are organized in a cascaded manner for joint training. In [6], a CycleGAN image enhancement module and SSD detection module are integrated into one framework, and the relevant detection information from the detector is applied to guide the optimization of the enhancement module in a direction conducive to detection tasks.

End-to-end frameworks can enhance the performance of detection tasks but introduce additional computational costs. DSNet [7] utilizes a dual-subnet structure in which the recovery subnet and detection subnet are connected in parallel, sharing a common block. During training, both subnets are trained jointly, but during the detection, only the detection subnet is used. Consequently, no additional computational cost is introduced. In JADSNet [8], a joint attention-guided dual-subnet network is introduced to address the problems in marine object detection through jointly learning image enhancement and object detection tasks. The detection subnet utilizes RetinaNet as the backbone to classify and locate the objects, and the image enhancement subnet shares the feature extraction layer with the detection subnet. Liu et al. [3] organized the detection branch and enhancement branch in a parallel manner, using enhanced images to guide the lower layers of the detection branch to learn lost details. This effectively improved the precision of detection while obtaining enhanced output with excellent visual appearance; however, excellent visual appearance does not guarantee optimal object detection performance.

## 3. Methods

### 3.1. Method Overview

**Figure 2:**
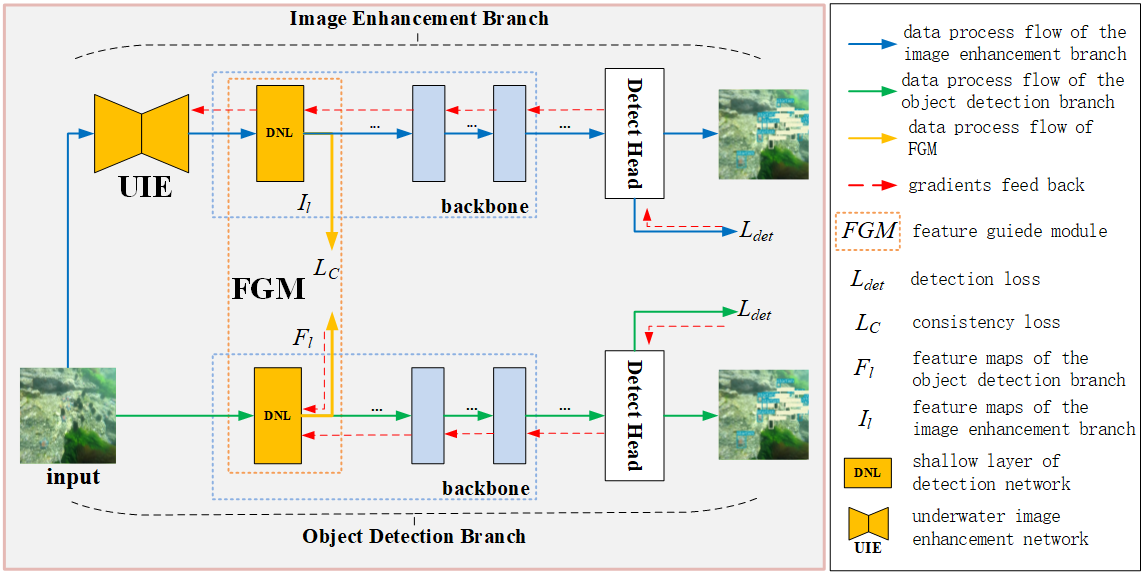
Overview of BG-YOLO framework.

The research presented in [3] demonstrates that extracting low-level features is essential for detection in visually degraded scenes. To address the issue of image degradation causing difficulties in object detection in complex underwater scenes, we propose a bidirectional-guided object detection framework, transferring information between image enhancement and object detection. As shown in Figure 2, the framework consists of three parts: an image enhancement branch, an object detection branch, and a feature-guided module. The image enhancement branch and object detection branch are organized in a parallel manner. During the training, the features extracted by the enhancement branch are used to guide the detection branch to learn the low-level features and more detailed object information beneficial to the detection tasks and thereby enhance the detection performance. During the tests, we removed the image enhancement branch and feature-guided module, performing detection with only the trained detection branch. Compared to the method proposed in [6], no additional computational cost is introduced in object detection because there is no cascaded image enhancement network in our proposed framework.

Different from what is proposed in [3], we cascade an image enhancement subnet with an object detection subnet as an image enhancement branch instead of utilizing a pre-trained image enhancement network. When training the image enhancement branch, the detection subnet guides the enhancement subnet towards optimization conducive to object detection tasks. Subsequently, when training the detection branch, the parameters in the image enhancement branch are fixed.

Differently from the feature-guided module in [3], our method extracts features from the shallow convolutional layer of the object detection branch and the backbone of the detection subnet in the image enhancement branch, respectively. With the constraint of the proposed consistency loss, the low-level features of the object detection branch gradually tend towards the low-level features of the image enhancement branch, which enables the detection branch to extract more detailed information about objects.

It is worth mentioning that, different from other joint optimization methods [3,5,7], the objective of our proposed method is better performance in underwater object detection. The visual appearance of the output of the enhancement branch is not taken into consideration.

### 3.2. Image Enhancement Branch

**Figure 3:**
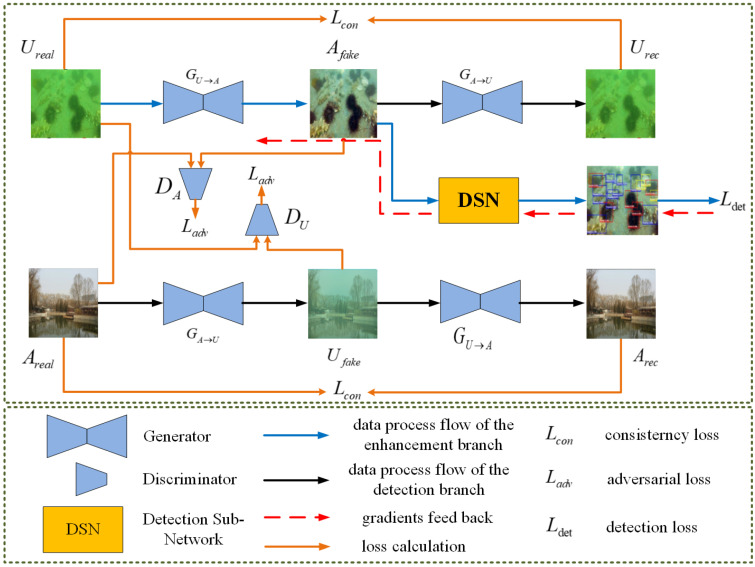
Overview of the image enhancement branch.

A cascade of the image enhancement subnet and object detection subnet is utilized as the image enhancement branch, which makes the image enhancement subnet more conducive to improving the performance of the object detection subnet. The structure of the image enhancement branch is shown in Figure 3, which consists of an image enhancement subnet and a detection subnet (DSN).

The image enhancement step utilizes generators similar to the CycleGAN structure, as introduced in [33]. CycleGAN is constructed by two generators, $G_{U\to A}$ and $G_{A\to U}$, and two adversarial discriminators, $D_{U}$ and $D_{A}$. The generator $G_{U\to A}$ transfers the degraded underwater images $U_{real}$ from the underwater domain to the in-air domain and consequently generates the output images $A_{fake}$. The other generator, $G_{A\to U}$, transfers in the opposite direction; to be precise, it transfers the underwater images that were previously transferred to the in-air domain back to the underwater domain, consequently generating the output $U_{rec}$. The adversarial discriminator $D_{A}$ prompts the generator $G_{U\to A}$ to transform the original underwater images $U_{real}$ into output that is difficult to distinguish from real clear in-air images $A_{real}$. Similarly, it prompts the generator $G_{A\to U}$ to transform real clear in-air images $U_{real}$ into output that is difficult to distinguish from underwater images $U_{real}$.

DSN is designed to describe the internal features and semantic information necessary for detection tasks, emphasizing more features beneficial for detection and transmitting the perception of the detector to the image enhancement subnet so that the potential output of the image enhancement subnet is more conducive to detection.

With the intention of making the features extracted by the subsequent feature-guided branch more comparable, DSN is implemented using YOLOv5s, constituting the image enhancement branch with the generator $G_{U\to A}$ of CycleGAN, as shown by the blue arrows in Figure 2 and Figure 3. The pre-trained generator $G_{U\to A}$ is utilized as the image enhancement module. Then, the DSN is pre-trained with the enhanced data to acquire fundamental knowledge of target classes. Finally, the framework is trained with the original underwater dataset with annotations to achieve appreciable detection performance.

### 3.3. Object Detection Branch

In the proposed framework, numerous existing detection networks can be used as the detection branch. The YOLO family of object detectors is widely used in underwater object detection. Some studies have shown that, for specific evaluation indicators and datasets, the performance of newer versions of the YOLO detector is not necessarily better than that of YOLOv5s [44]. YOLOv5s is an effective version for detecting underwater objects [45]. In this study, we selected YOLOv5s as the detection branch. For a detailed description of YOLOv5s, please refer to [46]. In the framework, the image enhancement branch is supposed to guide the optimization of the shallow layers of the backbone network in the object detection branch. As shown by the orange arrows in Figure 2, multiple low-level features in different scales are extracted from the shallow layers of the detection network. These low-level features extracted from the detection branch tend to be consistent with the corresponding low-level features of the enhancement branch under the guidance of the feature-guided branch, which makes the features of objects more salient for the detector and is conducive to improving the performance of object detection.

### 3.4. Feature-Guided Module

In images captured in an underwater environment, some features of objects are distorted or even obscured by the complex background, and the degradation of the images worsens this distortion. Therefore, these objects may be ignored by the network at the beginning. Ensuring that the shallow features of the detection network tend to be consistent with the image enhancement features that are conducive to object detection will make the features of the objects more prominent. Propagating these prominent features to the deeper layers of the object detection network can effectively improve the performance of object detection.

**Figure 4:**
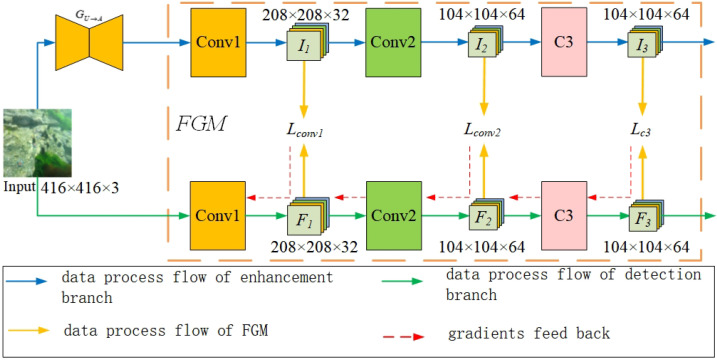
Overview of the feature-guided module.

The feature-guided module constrains the low-level features *F* extracted by the detection subnet of the object detection branch to converge to *I*, the low-level features extracted by the detection subnet of the image enhancement branch. An overview of its structure is shown in Figure 4. The input of the feature-guided module originates from two sources: the multi-level shallow feature mappings $F_{l},l\in \left\{1,\;2,\;3\right\}$ extracted by the detection subnet of the object detection branch and the multi-level shallow feature mappings $I_{l},l\in \left\{1,\;2,\;3\right\}$ extracted by the detection subnet of the image enhancement branch. $F_{l}$ and $I_{l}$ are extracted from the corresponding layer of the object detection subnet; both have the same dimension and similar semantic information. Subsequently, the consistency loss is minimized to make the feature mappings, $F_{l}$ and $I_{l}$, tend towards consistency. The enhancement subnet is fixed when training to constrain the shallow layers of the detection subnet to be optimized in a direction conducive to image enhancement, thereby obtaining more detailed information about the objects.

### 3.5. Loss Function

#### 3.5.1. Image Enhancement Loss

The image enhancement subnet is designed to generate clear in-air images from the input degraded underwater images. CycleGAN has two generators, $G_{U\to A}$ and $G_{A\to U}$, and two corresponding adversarial discriminators, $D_{U}$ and $D_{A}$. For the mapping $G_{U\to A}$ and its corresponding discriminator $D_{A}$, the adversarial loss can be mathematically expressed as follows:

(1) $$\begin{array}{cc} \;L_{GAN}\left(G_{U\to A},D_{A},X_{U},X_{A}\right)= & \\ \; & \;E_{x_{a}∼p_{data}\left(x_{a}\right)}\left[\log D_{A}\left(x_{a}\right)\right]\\ \; & \;+E_{x_{u}∼p_{data}\left(x_{u}\right)}[\log\left(1-D_{A}\left(G_{U\to A}\left(x_{u}\right)\right)\right] \end{array}$$

where mapping $G_{U\to A}$ is supposed to generate images $G_{U\to A}\left(X_{u}\right)$ similar to images in the in-air domain $X_{A}$ using images in the underwater domain $X_{U}$, and the discriminator $D_{A}$ is expected to distinguish between the generated in-air domain images $G_{U\to A}\left(X_{u}\right)$ and the real in-air domain images $X_{a}$. Similarly, for the mapping $G_{A\to U}$ and its corresponding discriminator $D_{U}$, the adversarial loss can be mathematically expressed as follows:

(2) $$\begin{array}{cc} \;L_{GAN}\left(G_{A\to U},D_{U},X_{A},X_{U}\right)= & \\ \; & \;E_{x_{u}∼p_{data}\left(x_{u}\right)}\left[\log D_{U}\left(x_{u}\right)\right]\\ \; & \;+E_{x_{a}∼p_{data}\left(x_{a}\right)}\left[\log\left(1-D_{U}\left(G_{A\to U}\left(x_{a}\right)\right)\right)\right] \end{array}$$

The cycle consistency loss prevents the learned mappings $G_{U\to A}$ and $G_{A\to U}$ from being contradictory. For each image $X_{u}$ from domain $X_{U}$, the forward cycle of image transfer is supposed to transform $X_{u}$ back into the original image $X_{u}$. Similarly, for each image $X_{a}$ from the domain $X_{A}$, the consistency loss [32] can be expressed as follows:

(3) $$\begin{array}{cc} \;L_{con}\left(G_{U\to A},G_{A\to U}\right)= & \\ \; & \;E_{x_{u}∼P_{data}\left(x_{u}\right)}\left[{∥G_{A\to U}\left(G_{U\to A}\left(x_{u}\right)\right)-x_{u}∥}_{1}\right]\\ \; & \;+E_{x_{a}∼P_{data}\left(x_{a}\right)}\left[{∥G_{U\to A}\left(G_{A\to U}\left(x_{a}\right)\right)-x_{a}∥}_{1}\right] \end{array}$$

The cycle consistency loss [47] is introduced to maintain the composition of the original image through extracting features in both high and low levels using VGG-16. The cycle consistency loss can be mathematically expressed as follows:

(4) $$\begin{array}{cc} \;L_{cp}\left(G_{U\to A},\;G_{A\to U}\right)= & \\ \; & \;∥\phi \left(x_{u}\right)-\phi \left(G_{A\to U}\left(G_{U\to A}\left(x_{u}\right)\right)\right){∥}_{2}^{2}\\ \; & \;+∥\phi \left(x_{a}\right)-\phi \left(G_{U\to A}\left(G_{A\to U}\left(x_{a}\right)\right)\right){∥}_{2}^{2} \end{array}$$

The total loss of image enhancement $L_{UIE}$ can be expressed as follows:

(5) $$\begin{array}{cc} \;L_{UIE}\left(G_{U\to A},G_{A\to U},D_{A},D_{U}\right)= & \\ \; & \;L_{GAN}\left(G_{U\to A},D_{A},X_{U},X_{A}\right)\\ \; & \;+L_{GAN}\left(G_{A\to U},D_{U},X_{A},X_{U}\right)\\ \; & \;+\lambda_{1}L_{con}\left(G_{U\to A},G_{A\to U}\right)\\ \; & \;+\lambda_{2}L_{cp}\left(G_{U\to A},G_{A\to U}\right) \end{array}$$

where $\lambda_{1}$ and $\lambda_{2}$ control the relative importance of the two losses.

#### 3.5.2. Object Detection Loss

Both the object detection module of the image enhancement branch and the object detection branch utilize the same structure as YOLOv5s and consequently use the same loss functions as the original YOLOv5s, as shown below:

(6) $$\begin{array}{cc} \;L_{det} & =a\cdot {\mathrm{LOSS}}_{obj}+b\cdot {\mathrm{LOSS}}_{loc}+c\cdot {\mathrm{LOSS}}_{cls} \end{array}$$

where ${LOSS}_{obj}$ is the confidence loss, a function of the probability that an object exists, more specifically, whether an object exists inside the predicted bounding box; ${LOSS}_{obj}$ is the classification loss, a binary cross-entropy function of the predicted probability that the object in a bounding box belongs to a class and the ground truth; and ${LOSS}_{loc}$ is a function measuring the difference between the predicted bounding boxes and the ground truth. a, b, and c are the weight factors, respectively.

#### 3.5.3. Consistency Loss

To measure the consistency of the low-level features of the object detection branch and the enhancement branch, we utilize the mean square error (MSE) function as the consistency loss. The MSE, convex and differentiable, is widely used in metrics in regression, pattern recognition, signal and image processing, etc. We use the MSE to measure the difference between the feature maps at the pixel level and attempt to minimize it. With consistency loss, the detection network is able to understand the subtle features of the distribution of objects.

The feature-guided consistency loss is expressed as follows:

(7) $$\begin{array}{c} L_{con}=\frac{1}{h\cdot w}\sum_{i=0}^{h-1}\sum_{j=0}^{w-1}{\left[F_{l}\left(i,j\right)-I_{l}\left(i,j\right)\right]}^{2} \end{array}$$

where *h* and *w* are the height and width of the feature map, and $F_{l}\left(i,j\right)$ and $I_{l}\left(i,j\right)$ represent the features from the l-th level of the object detection subnet and the image enhancement subnet, respectively. The full guided loss is mathematically expressed as follows:

(8) $$\begin{array}{cc} L_{FGM} & =\mu_{1}L_{con1}+\mu_{2}L_{con2}+\mu_{3}L_{c3} \end{array}$$

where $\mu_{l},l\in \left\{1,\;2,\;3\right\}$ is used to balance the consistency loss between the different feature layers $\left(F_{l},I_{l}\right),l\in \left\{1,\;2,\;3\right\}$.

#### 3.5.4. Total Loss Function

The image enhancement branch and object detection branch are trained separately. When training the image enhancement branch, the image enhancement subnet and object detection subnet are first trained separately; thus, the loss functions are, respectively, the image enhancement loss and object detection loss. Subsequently, only the object detection loss is used when jointly optimizing the image enhancement subnet.

When training the object detection branch, the parameters of the image enhancement branch are fixed, and the total loss of the object detection branch is defined as the sum of the detection loss $L_{det}$ and the consistency loss $L_{FGM}$, as shown below:

(9) $$\begin{array}{cc} L & =\eta_{1}L_{det}+\eta_{2}L_{FGM} \end{array}$$

where $\eta_{1}$ and $\eta_{2}$ are balance factors.

## 4. Experiments and Discussion

### 4.1. Datasets

We adopted publicly accessible datasets, Underwater Robot Picking Contest 2019 (URPC2019) and Underwater Robot Picking Contest 2020 (URPC2020), to evaluate the performance of our proposed object detection framework. The URPC datasets are widely used to evaluate the performance of object detection methods in underwater scenes, and can be downloaded from http://www.cnurpc.org, accessed on 11 November 2024.

The URPC2019 dataset contains 3765 images in the training set and 942 in the testing set, covering five classes of underwater targets: echinus, starfish, holothurian, scallop, and waterweeds. The images in the dataset present adverse characteristics including color deviation, blurriness, low contrast, clustered objects, and occlusion.

The URPC2020 dataset includes a training set consisting of 4200 randomly chosen images and a testing set consisting of 800 randomly chosen images. This dataset covers four different kinds of underwater targets: holothurian, echinus, scallop, and starfish.

### 4.2. Implementation Details

We implemented our framework using Python-3.8.10, Torch-1.10.0, with an Intel Xeon(R) Platinum 8255C CPU @ 2.50 GHz, 43 GB of memory, and an Nvidia GeForce RTX3090 (Santa Clara, CA, USA).

When training BG-YOLO, first, joint optimization was applied on the image enhancement branch, and then, the pre-trained model was used to guide the training of the object detection branch. The image enhancement subnet is a publicly released CycleGAN network model, and the detection subnet is a publicly released YOLOv5s model.

#### 4.2.1. Training of the Image Enhancement Branch

To train the image enhancement branch, the image enhancement subnet and object detection subnet were first separately trained. The joint optimization of the two followed.

When training the image enhancement branch, the training details in [6,32] were partly referenced. The images for training were from the dataset UIEB [48] and the dataset EUVP [49]. UIEB contains 890 pairs of images, with each pair containing one clear image and a corresponding blurred one. EUVP contains 11,435 degraded/clear images on various backgrounds. We randomly chose 400 unpaired degraded/clear images from UIEB and 600 from EUVP as the training set. The images were then scaled to 416 × 416. During the experiments, we found that when we chose the clear in-air image as the target domain image, the transferred image suffered from obvious artifacts, distortion, and checkerboard effects, severely affecting the performance of the detector. We finally chose the clear underwater images as the target domain. When training the image enhancement subnet, the Adam optimizer was used. We trained the subnet for 50 epochs, with batch size = 2, momentum $\beta_{1}=0.5$, learning rate lr = 1 × $10^{-4}$, and the weights $\lambda_{1}=5\times 10^{-5}$ and $\lambda_{2}=1$.

When training the detection subnet of the image enhancement branch, we chose the optimal configurations according to the test results referring to the training details in [6]. The URPC2019 and URPC2020 datasets were used. We first used the pre-trained enhancement subnet mentioned above to enhance the images from the URPC datasets, and then, the enhanced images served as the input of the detection subnet. The optimizer utilized was SGD. We trained the subnet based on the publicly released pre-trained model yolov5s.pt for 300 epochs, with batch size = 16, momentum = 0.937, learning rate lr = 1 × $10^{-2}$, lrf = 1 × $10^{-2}$, and weight decay = 5 × $10^{-4}$.

When performing joint optimization on the image enhancement branch, we chose the optimal configurations according to the test results referring to the training details in [6]. The URPC2019 and URPC2020 datasets were used. When training, the pre-trained enhancement subnet model and pre-trained detection subnet model mentioned above were first loaded. The optimizer used was SGD. We trained the branch for 300 epochs with batch size = 16, momentum = 0.937, learning rate lr = 1 × $10^{-3}$, lrf = 1 × $10^{-3}$, and weight decay = 5 × $10^{-4}$.

#### 4.2.2. Training of the Object Detection Branch

The object detection branch consisted of only one detection subnet. When training, the pre-trained model of the image enhancement branch obtained through joint optimization was loaded. The parameters of the enhancement branch were fixed, and optimization was only applied to the object detection branch. The URPC2019 and URPC2020 datasets were used. The optimizer used was SGD. We trained the branch for 300 epochs with batch size = 16, momentum = 0.937, learning rate lr = 1 × $10^{-2}$, lrf = 1 × $10^{-2}$, and weight decay = 5 × $10^{-4}$. The object detection branch adopted the publicly released pre-trained model yolov5s.pt.

#### 4.2.3. Comparison

To evaluate the performance of our proposed method, we reproduced the corresponding proposed algorithms according to the details in [3,6] for comparison. However, we utilized YOLOv5s as the object detection network in all these algorithms to control variables and facilitate comparison, which would not have affected the final conclusion.

### 4.3. Evaluation Indices

To comprehensively and objectively evaluate the performance of our proposed method, we used the mean average precision (mAP), recall, precision, F1-score, PR curve, and detection speed (FPS). When testing the detection speed, the batch size was set to 1.

### 4.4. Visualized Comparison

**Figure 5:**
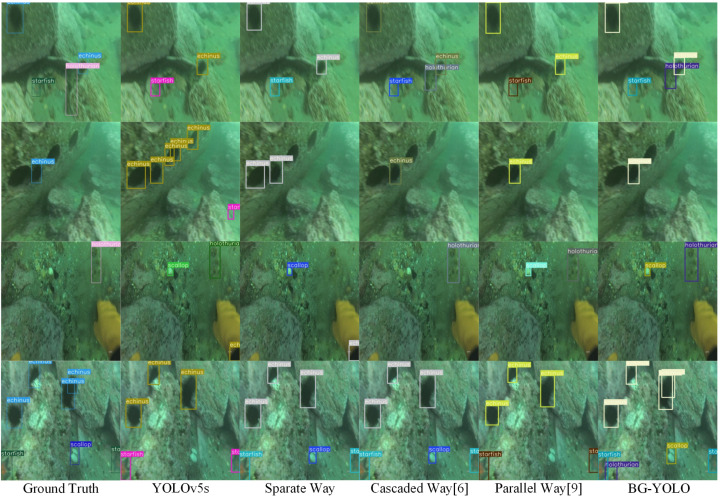
Visualized detection results for different methods on the URPC2019 dataset.

In this section, we first compare the visualized detection results for the original YOLOv5s, the separate way, the cascaded way, the parallel way, and the proposed BG-YOLO on the URPC2019 dataset. The visualized results in Figure 5 show that our proposed method can better detect clustered and occluded objects in degraded underwater images.

**Figure 6:**
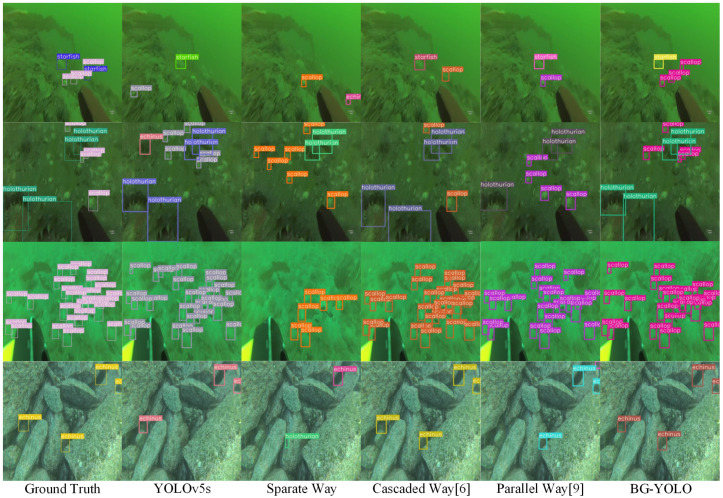
Isualized detection results for different methods on the URPC2020 dataset.

Figure 6 shows the visualized results for four images sampled from the URPC2020 dataset. It can be seen that BG-YOLO shows better detection performance in severely degraded underwater scenes, regardless of whether the objects are small, clustered, or occluded.

The visualized results demonstrate that BG-YOLO achieves the best performance among the approaches mentioned above. This is because our method optimizes the enhancement network in the way most conducive to detection when performing joint optimization, which alleviates the image enhancement’s performance impact on the detection task. When guiding the object detection branch, BG-YOLO makes the low-level features of the detection subnet tend to be more conducive to detection tasks, rather than focusing on the enhanced image itself.

### 4.5. Quantitative Comparison

#### 4.5.1. Results for URPC2019 Dataset

We utilized the third-party publicly released YOLOv5s as the basic network model. We conducted tests on the URPC2019 dataset, comparing the detection performance of the original YOLOv5s, the separate way, the cascaded way, the parallel way, and BG-YOLO. When testing, we first resized the images to 416 × 416, and the division of the dataset was the same as that of the original dataset, with 3765 images in the training set and 942 in the validation set (testing set). The cascaded way and parallel way are reproductions of the methods proposed in [3,6], respectively. The test results are shown in Table 1.

From Table 1, it can be seen that the mAP@0.5 of our proposed BG-YOLO is 78.4%, and the mAP@0.5–0.95 is 44.7%, marking improvements of 3.1% and 2.7%, respectively, compared to the baseline YOLOv5s. Compared to the separate method, cascaded method, and parallel method, BG-YOLO achieved improvements of 3.7% and 5.1%, 0.6% and 4.4%, and 1.5% and 2.6%, respectively. Additionally, the F1-score of BG-YOLO was also the best among the methods mentioned above, which demonstrates that BG-YOLO achieves an optimal balance between detection precision and recall. The analysis of the visualized detection results (Figure 5) shows that the original YOLOv5s achieves a higher recall rate than BG-YOLO; however, its false positive rate in detection is also higher. Additionally, the inference speed of BG-YOLO is also the fastest among these methods, reaching 130 fps, while the speed of the cascaded way is only 42 fps because of the computational cost incurred by combining UIE and UOD into one framework.

**Figure 7:**
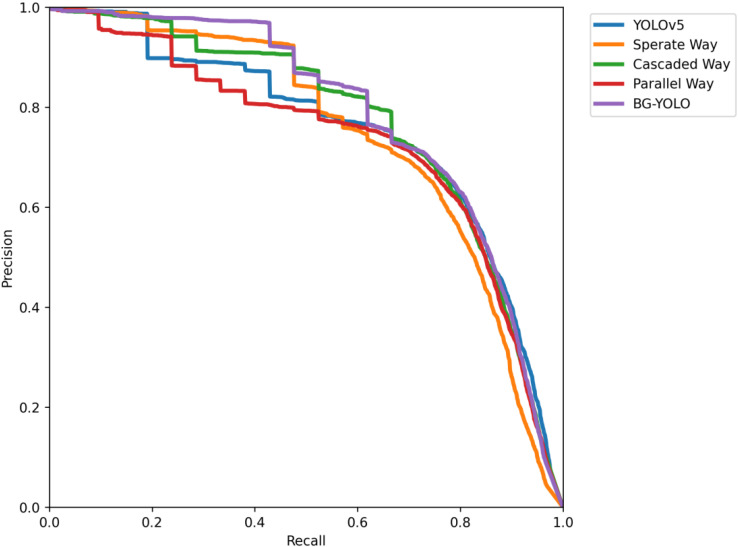
PR curve of the test results for URPC2019.

From Figure 7, it can be seen that the area enclosed by the PR curve of BG-YOLO (purple) and the coordinate axes is the largest, indicating that BG-YOLO achieves the best performance among these methods.

#### 4.5.2. Results for URPC2020 Dataset

We further conducted tests on URPC2020 in the same way as described in the previous section. The test results are shown in Table 2.

**Figure 8:**
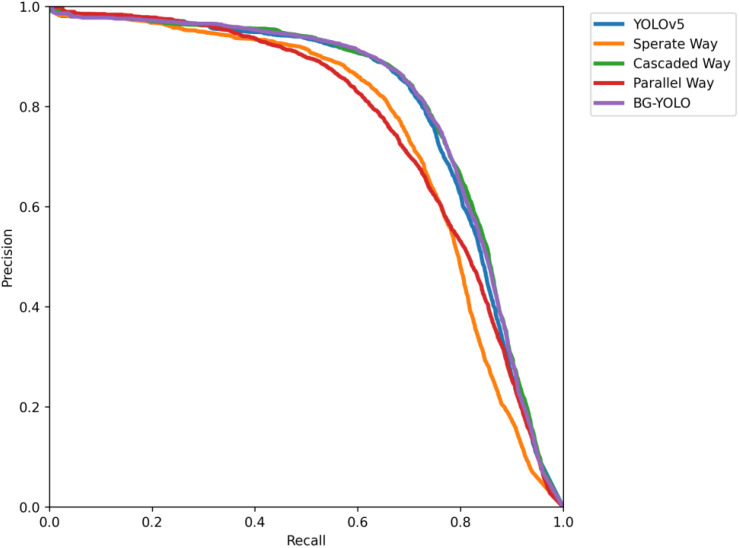
PR curve of the test results for URPC2020.

As is shown in Table 2, the mAP@0.5 of our proposed BG-YOLO is 80.2%, which is 0.7% higher than baseline, and 5.4% and 4.3% higher than those of the separate way and parallel way, respectively. Though slightly lower than the cascaded way (0.2%) in terms of mAP@0.5, BG-YOLO is three times as fast. The PR curves shown in Figure 8 also demonstrate the performance of BG-YOLO in comparison to other methods.

It is worth mentioning that the parallel approach reproduced with reference to [3] performed even worse than the original YOLOv5s. This is because we did not adopt a particular enhancement algorithm when reproducing it, which also demonstrates that the performance of the approach in [3] depends on the enhancement algorithm chosen.

#### 4.5.3. Comparison with Other Algorithms

We also compared the performance of BG-YOLO with that of other algorithms on the URPC2019 and URPC2020 datasets. The compared algorithms include YOLOv7, YOLOv8s, the algorithm in [6], and the algorithm in [3]. YOLOv7 and YOLOv8s both use pre-trained models and default training parameters published by third parties. We reproduced the algorithms in [3,6] and trained them according to the experimental details described in the literature. The experimental results are presented in Table 3.

From the test results shown in Table 3, it can be observed that BG-YOLO performed better than all the other algorithms on the URPC2019 dataset, with the mAP@0.5 and mAP@0.5–0.95 scores exceeding those of the compared algorithms by up to 5.3% and 3%, respectively. When the method proposed in this chapter was run on the URPC2020 dataset, its mAP@0.5 and mAP@0.5–0.95 were better than those in [6] and slightly lower than those in [3], but the inference speed of our method far exceeded that in [3]. The above data indicate that BG-YOLO can significantly improve the performance of object detection in low-quality underwater images. It is notable that, on the URPC2020 dataset, BG-YOLO’s mAP@0.5 and mAP@0.5–0.95 scores were lower than those of YOLOv7 and YOLOv8, which is mainly due to the advantages conferred by advanced object detection algorithms and does not affect the conclusions drawn from the algorithms in this chapter.

It is notable that the detection performance of the parallel organization method as shown in Table 1, Table 2 and Table 3 was even lower than that of the original YOLOv5s. Upon analyzing the enhanced dataset intended for guidance, it was observed that the enhanced images introduced numerous artifacts, noise, and checkerboard patterns, all of which negatively impacted the performance of the ultimate detection task. This suggests that the detection performance of the algorithm presented in [3] is constrained by the outcomes of image enhancement. In other words, the enhanced branch of BG-YOLO can guide the enhancement subnet to optimize in a direction more conducive to detection tasks, thus compensating for the shortcomings of the enhancement subnet without special consideration of the performance of the enhancement algorithm.

#### 4.5.4. Effects of Feature-Guided Layer

To evaluate the influence of different shallow convolutional layers $f_{1}$ and $I_{1}$, we tested the difference in guiding the training of BG-YOLO by extracting features $\left(F_{l},I_{l}\right),l\in \left\{1,\;2,\;3\right\}$ from the first convolutional layer conv1, the second convolutional layer conv2, and the C3 module of the enhancement branch and the corresponding detection network in the detection branch on the URPC2019 dataset. The results are presented in Table 4. In these experiments, the weight factors of the detection loss $L_{det}$ and consistency loss $L_{FGM}$ were $\lambda_{1}=\lambda_{2}=1.0$, and the weight factors of $L_{con1}$, $L_{con2}$, and $L_{C3}$ were $\mu_{1}=\mu_{2}=\mu_{3}=1.0$.

As shown in Table 4, each combination of the feature-guided layers presented above contributes to improving the detection performance, which indicates that guiding the low-level features of the detection network towards image enhancement is conducive to detection tasks. When using only the features of the first convolutional layer for guidance, the best detection performance is achieved. In degraded underwater scenes, the main reason for the decline in object detection performance is that the degraded underwater images lack detailed features conducive to object detection. Additionally, deeper convolutional layers focus more on semantic information, which does not significantly contribute to improving object detection performance.

#### 4.5.5. Effects of Different η

According to formula (9), the values of $\eta_{1}$ and $\eta_{2}$ balance the effects of the object detection branch and the enhancement branch. We fixed $\eta_{1}$ and tested the contribution of the guiding of the enhancement branch with different values of $\eta_{2}$. Considering the results of the previous section, only the shallowest convolutional layer was used for feature guidance. The dataset used in these experiments was URPC2019. The results are presented in Table 5.

The results presented in Table 5 indicate that it is favorable for detection tasks when $\eta_{2}$ varies from 0.01 to 1.0, and when $\eta_{2}=0.05$, the model reaches the highest mAP@0.5, 78.4%.

## 5. Conclusions

The degradation of images is a significant challenge for underwater object detection tasks. Using image enhancement methods to improve the visual effects of underwater images is not necessarily conducive to improving detection performance. To address the above issues, we propose a bidirectional guided object detection method, which combines the advantages of both a cascaded organization and parallel organization of the image enhancement and object detection networks. More specifically, we organize the enhancement branch and detection branch in parallel, and the enhancement branch is a combination of the enhancement subnet and detection subnet. The detection branch contains only one detection subnet. The feature-guided module guides the low-level features to be optimized towards the corresponding low-level features of the enhancement branch, in order to make the detection branch sensitive to the quality of the images and object detection. During the inference, the enhancement branch and feature-guided module are removed, and only the detection branch is used. Extensive experiments demonstrate that our proposed framework can significantly improve the precision of underwater object detection without introducing additional computational costs, which demonstrates the effectiveness of our proposed method.

Underwater images are easily degraded by complex underwater environments, which is not conducive to subsequent object detection tasks. Image visual quality evaluation and underwater image enhancement require further research to improve image processing in object detection.

## Figures and Tables

**Table 1:** Test results for URPC2019.

Method	mAP@0.5 (%)	mAP@0.5–0.95 (%)	Recall (%)	F1-Score (%)	Precision (%)	FPS (fps)
YOLOv5s	75.3	42.0	72.6	74.0	74.8	130
Separate Way	74.7	39.6	68.6	75.0	83.4	130
Cascaded Way	76.9	42.8	72.7	76.0	81.0	42
Parallel Way	73.1	41.7	66.3	72.0	78.7	130
BG-YOLO	78.4	44.7	70.1	77.0	88.3	130

**Table 2:** Test results for URPC2020.

Method	mAP@0.5 (%)	mAP@0.5–0.95 (%)	Recall (%)	F1-Score (%)	Precision (%)	FPS (fps)
YOLOv5s	79.5	44.9	73.8	78.0	83.7	132
Separate Way	74.8	40.3	69.1	75.0	81.1	132
Cascaded Way	80.4	44.8	75.7	79.0	82.0	42
Parallel Way	75.9	38.4	68.1	73.0	79.7	132
BG-YOLO	80.2	44.6	75.2	79.0	82.7	132

**Table 3:** Performance comparison of different algorithms on URPC2019 and URPC2020 datasets.

Method	URPC2019-mAP@0.5 (%)	URPC2019-mAP@0.5–0.95 (%)	URPC2020–mAP@0.5 (%)	URPC2020-mAP@0.5–0.95 (%)
YOLOv5s	75.3	42.0	79.5	44.9
Algorithm in [6]	76.9	42.8	80.4	44.8
Algorithm in [3]	73.1	41.7	75.9	38.4
YOLOv7	77.5	43.2	81.3	44.1
YOLOv8	77.9	45.5	81.8	47.6
BG-YOLO	78.4	44.7	80.2	44.6

**Table 4:** Contribution of feature-guided layers to detection precision; “√” indicates that the operation has been performed.

conv1	conv2	C3	mAP@0.5 (%)	mAP@0.5–0.95 (%)
			75.3	42.0
√			78.0	43.8
	√		77.7	44.2
		√	77.0	43.8
√	√		76.0	43.5
√		√	76.8	41.6
	√	√	76.6	43.5
√	√	√	76.6	43.6

**Table 5:** Detection results with different $\eta_{2}$.

$\eta_{2}$	mAP@0.5	mAP@0.5–0.95
1.0	78.0	43.8
0.5	77.4	44.6
0.1	77.6	43.6
0.05	78.4	44.7
0.01	76.6	44.8

## Data Availability

The raw data supporting the conclusions of this article will be made available by the authors on request.
